# The digital divide: Racial disparities in adoption and utilization of health information technology among patients with lymphoid cancers

**DOI:** 10.1002/cam4.6454

**Published:** 2023-08-17

**Authors:** Sikander Ailawadhi, Meghna Ailawadhi, Navnita Dutta, Ricardo D. Parrondo, Vivek Roy, Taimur Sher, Mizba Baksh, Ahsan Rasheed, Saurav Das, Andre J. Fernandez, Aneel Paulus, Asher A. Chanan‐Khan

**Affiliations:** ^1^ Division of Hematology‐Oncology Mayo Clinic Jacksonville Florida USA; ^2^ Division of Cancer Biology Mayo Clinic Jacksonville Florida USA

## Abstract

**Introduction:**

Health information technology (HIT) has the potential to improve healthcare delivery and engagement. Studying racial‐ethnic disparities in HIT engagement will help understand and overcome challenges to healthcare utilization.

**Methods:**

We undertook a patient‐reported survey among patients with lymphoid malignancies at two campuses of Mayo Clinic, Florida to explore HIT‐related disparities. Variables between Whites and non‐Whites, and non‐Whites from the two campuses were compared.

**Results:**

The survey was completed by 1004 respondents, with 71% whites, 27% non‐Whites (race‐ethnicity not reported by 2%). Non‐Whites included 30% responders at the main campus and 64% at an inner‐city campus. Whites were significantly older and had higher education, while non‐Whites had lesser access to a computer. Only 51% of non‐Whites were registered to use electronic medical records (EMR) as compared to 72% Whites (*p* < 0.001) and significantly lesser number of non‐Whites even knew that EMR existed (81% vs. 92%, *p* < 0.001). Encouragingly, a higher number of non‐Whites wanted to engage in EMR. Non‐Whites from the main campus were older, more educated and had more access to a computer as compared to those from the inner‐city campus. Similar disparate factors were noted among minorities from the two campuses, suggesting impact of socioeconomic backgrounds on EMR usage among non‐Whites. Linguistic barriers were more striking among inner‐city campus non‐Whites.

**Conclusions:**

Non‐Whites continue to struggle with suboptimal utilization of the healthcare system and barriers related to integration in HIT, including disparities representing socioeconomic differences. Efforts need to be made at several levels to help racial‐ethnic minorities overcome awareness, access, and linguistic barriers to HIT utilization.

## 1 INTRODUCTION

Health information technology (HIT) has fast become a cornerstone of the healthcare delivery landscape. The various aspects of HIT including electronic health records (EHR), computerized provider order entry (CPOE), clinical decision support systems (CDSSs), physician notes, visit, and discharge summaries, etc. play a key role in transforming and augmenting the standard of care for patients, improving patient safety, chronic disease management, and coordination of information sharing during transitions in care.^1^, ^2^ The integration of these key HIT elements can lead to optimal shared decision making for patients and has also led to evidence‐based measures which are basis of quality outcomes as well as reimbursement models in the current healthcare sector.^3^

Data suggests that 57% of U.S. physicians and 92.6% of European general practitioners use HIT in clinical practice with EHR systems.^4^, ^5^, ^6^ While there has been an increase in HIT adoption among healthcare practices and professionals, its optimal benefit can only be realized with patient involvement and active participation. Nevertheless, the adoption of HIT among patients for their healthcare needs is quite low in the United States, according to some estimates, being a dismal 37%.^7^, ^8^ In recent years, the COVID‐19 pandemic has significantly transformed how healthcare is delivered, and how it is accessed by the patients.^9^ Now, more than ever, there is an unprecedented need for patients to engage digitally with the healthcare system, the so‐called digital health literacy, so that they can get access to and benefit from timely, evidence‐based care.^9^ This could be for a variety of healthcare needs including routine preventative care, acute or chronic ongoing healthcare conditions, or the myriad of growing health‐related issues being uncovered by a pandemic‐stricken society for example, mental health concerns.^10^

With time, the U.S. demographic mix has evolved, including an increasing proportion of non‐white racial groups, frequently termed racial minorities. Indeed, by 2050, racial minorities are forecasted to comprise approximately 50% of the U.S. population.^11^ With this shifting demographic, the impact of sociodemographic factors on healthcare access and utilization and the disparities that racial minorities face, have become evident, and are an important focus of research.^12^ A facet of these disparities includes what has been termed the digital‐divide, a disparity in engagement with HIT for patients of racial minorities, and other vulnerable populations.^13^ Prior studies have shown that racial minorities do not actively engage in the usage of HIT tools. This may impact their outcomes, and is an area of concern and active investigation by the Advisory Commission on Consumer Protection and Quality in the Health Care Industry.^14^, ^15^ Furthermore, the complex and interdisciplinary care needed for optimal outcomes in diagnoses such as cancer may be significantly hampered by lack of shared decision making and patient engagement stemming from the digital divide.^16^ Prospective studies in large cohorts to elucidate the digital divide among racial minorities from different sociodemographic backgrounds, and factors that may be addressed to narrow this disparity, are lacking. We undertook such a study to evaluate disparities in HIT adoption among a large cohort of cancer patients and their caregivers from different institutions, representing socio‐demographically distinct racial groups, and explored mitigation strategies.

## 2 METHODS

This study consisted of a self‐reported, anonymous, paper‐based questionnaire in English language. The questionnaire consisted of 21 questions addressing attitudes towards HIT, current understanding, and usage of HIT and any unmet needs regarding access to HIT. The questionnaire was administered prospectively to adult (≥18‐year‐old) patients with lymphoid malignancies and their caregivers seen at the Mayo Clinic campuses in Jacksonville, FL including the Main Campus (4500 San Pablo Rd., Jacksonville FL), and an inner‐city campus location (Ascension St. Vincent's Riverside, 1 Shercliff Way, Jacksonville FL). While the two sites were both affiliated, the patient population was socio‐demographically distinct due to the differences in prerequisite insurance requirement for receiving care at the two sites (e.g., no Medicaid patients seen at the Main Campus). Patients at both sites had access to an electronic medical record (EMR) system, although the two systems were different. While the local catchment area was similar, the main campus also attracted patients from regional, national, and international locations. In addition to HIT‐related questions, the questionnaire also included demographic variables including age, gender, race‐ethnicity (White vs. non‐White) and education (high school graduate or less, vs. some college degree, vs. bachelor's degree or more). Among those who were registered to access EMR, frequency of EMR access was scored as at least once per week, at least once per month, rarely (once in 3–4 months) or never. In addition to a comparison of White versus non‐White responders, an analysis was done to compare responses from non‐White patients from the two campuses. Categorical and continuous variables among groups were compared using chi‐squared test and Mann–Whitney *U*‐test, respectively with a *p* value ≤0.05 considered statistically significant.

This study was IRB exempt as all the responses obtained from patients were anonymous and voluntary without collecting any protected health information. Data were analyzed as an aggregate for the whole group.

## 3 RESULTS

**FIGURE 1:**
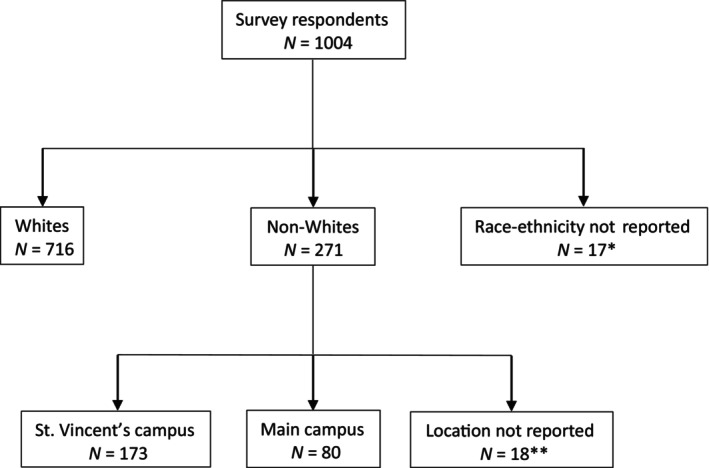
Consort diagram. Various respondent cohorts included in the respective statistical analyses for race and for non‐Whites by location. ^*^Respondents not included in analysis by race. ^**^Respondents not included in analysis for non‐Whites by location.

The survey was completed by 1004 individuals at Mayo Clinic, Florida at the two sites (Main Campus and St. Vincent Hospital). The HIT questionnaire respondents included 716 (71%) Whites, 271 (27%) non‐Whites, and 17 (2%) respondents who chose not to report race/ethnicity (Table 1; Figure 1). For statistical analysis, those without known race/ethnicity were not included. Whites were significantly older (IQR: 60–77 years) as compared to non‐Whites (IQR: 55–70 years) (median 66 years vs. 62 years; *p* < 0.001) and had a higher education level (60% undergraduate level or higher among Whites vs. 52% in non‐Whites; *p* = 0.02). Regarding access to technology, there was no statistically significant difference in access to a smartphone among Whites and non‐Whites, although Whites had access to a computer at work or home more frequently as compared to non‐Whites (92% vs. 84%; *p* < 0.001). EMR utilization and access related questions revealed that Whites were significantly more frequently aware that they could access medical records electronically (92% vs. 81%; *p* < 0.001) and a higher number of Whites were registered to use EMR via HIT as compared to non‐Whites (72% vs. 51%; *p* < 0.001). A significantly higher proportion of non‐Whites reported never using EMR, despite being registered for it (13% vs. 4%; *p* < 0.001). However, despite this lower rate of EMR utilization by non‐Whites, they were significantly more frequently interested in learning how to access the EMR electronically (43% vs. 36%; *p* = 0.04). While this survey was administered only in English language, and we did not obtain specific language preferences from respondents, majority of both Whites (57%) and non‐Whites (60%) thought it would be preferable if the EMR was in their primary language.

**TABLE 1:** Demographics and health IT usage of survey respondents.

Questionnaire element	White	Non‐White	*p* value
	*N* = 716 (71.3%)	*N* = 271 (27%)	
Median age (years)	66	62	<0.001
Education			
High school or less	185 (26%)	80 (30%)	0.20
Undergraduate or more	429 (60%)	140 (52%)	0.02
IT device			
Have a smartphone	634 (89%)	229 (85%)	0.08
Have a computer at home or work	658 (92%)	228 (84%)	<0.001
IT usage for non‐health purposes			
Pay bills	448 (62%)	161 (59%)	0.10
Financial/banking	485 (68%)	167 (62%)	0.46
Send and receive emails	580 (81%)	195 (72%)	0.23
Connect to social media	426 (59%)	160 (59%)	0.61
Buy tickets to travel	385 (54%)	132 (49%)	0.81
EMR access/usage			
Do you know you can access medical records electronically? (yes)	657 (92%)	220 (81%)	<0.001
Have you registered to access the EMR? (yes)			
If registered to access EMR, how often do you use it? (never)	512 (72%)	137 (51%)	<0.001
Are you interested in knowing how to access EMR electronically? (yes)			
	19 (4%)	18 (13%)	<0.001
	255 (36%)	117 (43%)	0.04
Factors to increase EMR access from home			
If it is in Spanish	4 (0.6%)	15 (6%)	0.38
If better access to computer	20 (3%)	24 (9%)	0.26
If someone can explain how to use it	70 (10%)	47 (17%)	0.002
If someone can explain the results	42 (6%)	39 (14%)	<0.001

Abbreviations: EMR, electronic medical records; IT, information technology; NS, not significant.

We explored reasons for why respondents did not access EMR for their healthcare needs more frequently. There were several reasons which non‐Whites reported more frequently for why they did not access EMR as compared to Whites including not knowing how to access EMR, not being able to understand the medical records, having a language barrier with the medical records, not being comfortable with computer/smartphone usage, forgetting to log on to the EMR, and not having sufficient time in the daily routine to log on to EMR, but these did not reach statistical significance. The reason for not increasing EMR usage which did reach statistical significance was due to not knowing that they could even access the EMR to get health records (18% for non‐Whites vs. 5% for Whites; *p* < 0.001). The only one reason that Whites cited more frequently for not accessing EMR usage as compared to non‐Whites was that they trusted the doctor to tell them everything and did not care about logging on to get the records themselves, but this was not statistically significant. To explore if there were differences in IT usage behavior among Whites and non‐Whites for purposes other than for healthcare needs, we asked the frequency with which respondents from both these groups performed tasks such as sending/receiving emails, doing travel bookings online, financial interactions, or accessing social media. None of these online behaviors were significantly different between Whites and non‐Whites.

We asked the respondents what factors could make them increase EMR usage. Several of the questions showed a numerical difference but did not reach statistically significant including preference of EMR to be in Spanish (6% for non‐Whites vs. 0.55% for Whites; *p* = 0.38) and increased access to computers (9% for non‐Whites vs. 3% for Whites; *p* = 0.26). But there were some questions which showed a statistically significant difference in the response among Whites and non‐Whites. Non‐Whites mentioned a higher likelihood to increase EMR use if someone explained how to use it (17% vs. 10% among Whites; *p* = 0.002) or if someone could explain test results to them (14% vs. 6% for Whites; *p* < 0.0001).

Among non‐Whites, a higher number (*n* = 173; 64%) were from St. Vincent's campus as compared to the main campus (*n* = 80; 29%), while (*n* = 18; 7%) did not specify the location they received care at (these 7% were not included in the comparative analysis of non‐White responders from various sites) (Figure 1). There were significant differences in non‐Whites from the two campuses, suggesting differences based on the patient's location of care, a surrogate of the socioeconomic background (Table 2). Non‐Whites at the main campus were significantly older and were more educated. IT device access was also different between the two campuses. Non‐Whites from the main campus had a significantly higher access to computers at home or work, although no statistically significant difference in smartphone access was noted. Minorities at the main campus were significantly more aware of the HIT‐based current EMR system and were registered to use the current patient EMR portal (93% vs. 75% and 76% vs. 38%, respectively; both *p* < 0.001). A higher proportion of non‐Whites from the inner‐city campus admitted to never using the EMR portal despite being registered on it as compared to at the main campus (22% vs. 7%; *p* = 0.018). Non‐Whites at the inner‐city campus were more frequently interested in learning how to access the EMR (47% vs. 33%; *p* = 0.036). A higher proportion of inner‐city non‐Whites reported that they were interested in learning how to access the EMR and increase their usage if optimally educated or if the test results were explained to them, but these differences did not reach statistical significance. A higher proportion of inner‐city non‐White respondents noted that it would be helpful if the EMR was in their primary language (66% vs. 48%; *p* = 0.006). Exploring factors that might increase EMR usage among patients showed several factors that had a numerical difference between main campus and inner‐city non‐whites but none that reached statistical significance.

**TABLE 2:** Demographics and health IT usage of non‐Whites from academic (Mayo Main Campus) and Inner City (St. Vincent's, Riverside) campus.

Questionnaire element	Non‐whites at main campus	Non‐whites at inner city site	*p* value
	*N* = 80	*N* = 173	
Median age (years)	67.5	57	<0.001
Education			
High school or less	21 (26%)	59 (34%)	0.20
Undergraduate or more	53 (66%)	81 (47%)	0.005
IT device			
Have a smartphone	72 (90%)	142 (82%)	0.10
Have a computer at home or work	75 (94%)	135 (78%)	0.001
EMR access/usage			
Do you know you can access medical records electronically? (yes)	74 (93%)	130 (75%)	<0.001
Have you registered to access the EMR? (yes)			
If registered to access EMR, how often do you use it? (never)	61 (76%)	66 (38%)	<0.001
Are you interested in knowing how to access EMR electronically? (yes)			
	6 (7%)	38 (22%)	0.018
	26 (33%)	81 (47%)	0.036
Will it help if it was in your primary language (yes)	38 (48%)	114 (66%)	0.006

Abbreviations: EMR, electronic medical records; IT, information technology; NS, not significant.

## 4 CONCLUSIONS

The American Recovery and Reinvestment Act (ARRA) of 2009, the Health Information Technology for Economic and Clinical Health (HITECH) Framework, Patient Protection and Affordable Care Act of 2010 (ACA) have facilitated the implementation of HIT in health care.^17^, ^18^ Increasing reports regarding the prevalence of health care disparities by race, ethnicity, education, language, demographic, and socio‐economic status suggest the uneven design of the U.S. health care system and how these disparities may affect patient's utilization of healthcare and their outcomes.^19^, ^20^, ^21^ While healthcare disparities between whites and racial minorities are multifactorial, it has been postulated that if carefully designed and implemented, HIT may have the ability to overcome some of the disparities that are widespread within our healthcare fabric.^18^ Yet, the digital divide between racial groups has the potential to further accentuate these disparities, and rather than being a solution, being additive to the problem itself.

Our study was done in a large number of patients with lymphoid malignancies at an academic center, with ample representation of a non‐White population. This overcomes the frequent issue related to healthcare disparities research, where Whites may still be the overwhelmingly large study population, hence leading to underrepresentation of the minorities, and leading to results which may not be generalizable.^22^ While it has been reported in several studies that non‐Whites may have a lower socioeconomic status (SES) and education level in general, finding the same in a homogenous population of patients at a large academic center underscores a societal issue which may sometimes be lost in a national‐level analysis. It has been reported that a high number of individuals within racial minorities have smartphones, but engagement with patient portals versus health apps may be disparate.^23^, ^24^ We noticed as well that while Whites and non‐Whites owned smartphones with similar rates, computer ownership, and use was significantly different among the two groups. As the capabilities and functionality of HIT and EMR portals may be different in mobile versus fixed point of care device, understanding this aspect is important so that specific strategies for the mode of use may be developed.^24^ A significantly higher proportion of non‐Whites were not aware that they could access health records electronically, were registered to use EMR, or even if registered, never used the EMR. A prior, smaller study in the VA system has shown some sociocultural issues related with lower EMR utilization by African‐Americans.^25^ In our study, it was very encouraging to see that a higher proportion of non‐Whites were interested in learning about the EMR as compared to whites, although this may be due to a larger existing knowledge gap among the non‐White cohort. We had set up our survey on the hypothesis that EMR utilization by non‐whites would be lesser and so, we explored what could increase EMR utilization by those who were not yet on board with it. Respondents mentioned that having someone explain how to use the EMR and the test results would make them use it more. This helps identify specific knowledge gaps that might be barriers for why some patients, especially racial minorities may not access HIT.

We addressed an aspect of racial disparities in healthcare, which is frequently overlooked – that even among a racial group, patients may belong to different socioeconomic strata and hence, may get affected by the disparities differently. Comparing non‐Whites from two different clinical settings in the same city, we were able to explore the attitudes of non‐Whites from different SES towards HIT and EMR usage. The further lower education level and lesser access to computers among inner‐city non‐Whites may help develop awareness and implementation strategies for better HIT adoption in this population, where the gaps may be most pronounced. Inner‐city non‐Whites were significantly less aware of HIT and were not enrolled for EMR utilization in higher numbers. Even if enrolled, they were much more likely to report not using EMR as compared to non‐Whites at the main campus. This could be influenced by the patient's relationship with their healthcare provider and team, who are the most frequent source of information related to healthcare and means to access it. Certainly, the demands on practice, healthcare provider cohort and patient characteristics would be distinct between the two sites but were not gathered within the confines of this study. We do feel that analyzing patient responses between the two sites helps understand some of this variability. Despite all these, a significantly higher proportion of inner‐city non‐Whites were interested in knowing more about EMR. We explored an impact of language barriers and found that inner‐city non‐Whites reported it would help if the EMR was in their primary language, suggesting there may be an impact of language services in the lesser adoption of HIT. This may suggest the void of widespread Spanish capabilities of most EMR systems in the US. Majority of Whites in our study were at the main campus, so we were unable to compare Whites between the two campuses, which may bring about systemic differences between populations in the respective catchment areas of the two locations.

This study does have some limitations, which provide us with insights into the design of future studies. As this is a questionnaire‐based study relying on self‐reported information, it is vulnerable to response bias favoring socially desirable responses. Furthermore, this study primarily addresses specialty care experiences within an academic medical center and the data were not collected from patients receiving primary care, which may limit the scope of responses for the survey. Still, the fact that these were all cancer patients with lymphoid malignancies makes them somewhat homogenous in their healthcare experiences and gathering data from two distinct sites; one representing an academic center and the other a relatively community‐based practice, provides sufficient breadth of responses to evaluate patient behavior by sociodemographic categories such as race. We attempted to collect specific race (African American, Asian, White, other) and ethnicity (Hispanic or non‐Hispanic) data but as this was self‐reported and not mandated to ensure respondent comfort, we were not able to obtain granular race‐ethnicity data and were only able to analyze responses based on White/non‐White. Nevertheless, this study provides insights into important and never previously studied issues, such that future studies for analyses by specific race and ethnicity can be designed. Our study did not incorporate individual level of proficiency with technology which older adults use most frequently for managing their health. But we did ask for other technology uses, for example, accessing social media, emails, shopping etc., which can serve as surrogates for technology use proficiency in respondents, and understand their behavioral preferences related to technology use. Our study did not explore the socioeconomic status of survey respondents, thus limiting our understanding of the impact of income level and other socioeconomic factors on HIT accessibility, which are most likely co‐dependent. This is being planned for a future study where more specific social determinant of health and access data collection will be attempted. This study used cross‐sectional data which hinders claims of causality. Longitudinal data would be more useful for examining the causes of disparities and how health information technology use changes over time. Our current analysis paves the way for such a longitudinal study, which would certainly be more resource intensive.

Not only is there a need for development of universal, uniform HIT infrastructure across the US that is tailor‐made to address the healthcare needs of patients, but the systems should also be accepted, adopted, and implemented across the cancer health ecosystem. The current study identifies significant gaps in HIT adoption and utilization among racial minorities and suggests redesigning strategies, which will improve the engagement of underrepresented groups who traditionally struggle to derive optimal utilization of healthcare advances. HIT has been proposed to achieve shared decision making, provide patients and their caregivers with better ownership of their healthcare utilization as well as efficiently implement evidence‐based medicine. Identifying gaps and utilizing strategies to bridge them will help not only with a more widespread and active utilization of HIT among all patient groups, but it will also help prepare a more engaged patient pool, where hopefully healthcare disparities are less prevalent. Future prospective studies with a diverse patient participation from multiple institutions will help confirm our findings also uncover any variability in patient behavior towards HIT in different clinical settings. This will hopefully help bridge the digital divide on a larger scale in the United States.

## AUTHOR CONTRIBUTIONS

**Sikander Ailawadhi:** Conceptualization (lead); supervision (lead); writing – original draft (lead); writing – review and editing (lead). **Meghna Ailawadhi:** Conceptualization (supporting); supervision (supporting); writing – original draft (supporting); writing – review and editing (supporting). **Navnita Dutta:** Supervision (supporting); writing – original draft (supporting); writing – review and editing (supporting). **Ricardo D Parrondo:** Conceptualization (supporting); writing – original draft (supporting); writing – review and editing (supporting). **Vivek Roy:** Conceptualization (supporting); writing – original draft (supporting); writing – review and editing (supporting). **Taimur Sher:** Conceptualization (supporting); writing – original draft (supporting); writing – review and editing (supporting). **Mizba Baksh:** Supervision (supporting); writing – original draft (supporting); writing – review and editing (supporting). **Ahsan Rasheed:** Supervision (supporting); writing – original draft (supporting); writing – review and editing (supporting). **Saurav Das:** Supervision (supporting); writing – original draft (supporting); writing – review and editing (supporting). **Andre J Fernandez:** Conceptualization (supporting); writing – original draft (supporting); writing – review and editing (supporting). **Aneel Paulus:** Conceptualization (supporting); writing – original draft (supporting); writing – review and editing (supporting). **Asher A Chanan‐Khan:** Conceptualization (supporting); supervision (supporting); writing – original draft (supporting); writing – review and editing (supporting).

## FUNDING INFORMATION

No research support was utilized for this research.

## Data Availability

Data sharing is not applicable to this article as no new data were created or analyzed in this study.
